# Transient Mechanical Response of Lung Airway Tissue during Mechanical Ventilation

**DOI:** 10.3390/bioengineering3010004

**Published:** 2016-01-05

**Authors:** Israr Bin Muhammad Ibrahim, Parya Aghasafari, Ramana M. Pidaparti

**Affiliations:** 1 Graduate Student, College of Engineering, University of Georgia, 597 DW Brooks Drive, Athens, GA 30602, USA; israr@uga.edu (I.B.M.I.); parya.aghasafari@uga.edu (P.A.); 2 College of Engineering, University of Georgia, 132A Paul D. Coverdell Center, Athens, GA 30602, USA

**Keywords:** mechanical strains, lung airway, mechanical ventilation, finite element analysis

## Abstract

Patients with acute lung injury, airway and other pulmonary diseases often require Mechanical Ventilation (MV). Knowledge of the stress/strain environment in lung airway tissues is very important in order to avoid lung injuries for patients undergoing MV. Airway tissue strains responsible for stressing the lung’s fiber network and rupturing the lung due to compliant airways are very difficult to measure experimentally. Multi-level modeling is adopted to investigate the transient mechanical response of the tissue under MV. First, airflow through a lung airway bifurcation (Generation 4–6) is modeled using Computational Fluid Dynamics (CFD) to obtain air pressure during 2 seconds of MV breathing. Next, the transient air pressure was used in structural analysis to obtain mechanical strain experienced by the airway tissue wall. Structural analysis showed that airway tissue from Generation 5 in one bifurcation can stretch eight times that of airway tissue of the same generation number but with different bifurcation. The results suggest sensitivity of load to geometrical features. Furthermore, the results of strain levels obtained from the tissue analysis are very important because these strains at the cellular-level can create inflammatory responses, thus damaging the airway tissues.

## 1. Introduction

Patients with respiratory problems whose lungs are compromised are treated with Mechanical Ventilation (MV) to assist them with breathing. MV can cause injury to airway lung tissue resulting from air pressure, and may eventually also cause damage to the lungs [1,2]. In general, the incidence of respiratory failures resulting from ventilator-associated lung injury (VALI) [3,4,5] is increasing due to the fact that there are still unanswered questions with regards to the transmission of mechanical forces into lung tissues resulting from MV. Mechanical aspects of VILI have been reported in past works, such as [3,4,5]. From these studies, ventilator management practices have been developed, such as maintaining positive end-expiration pressure. Despite this development, MV-induced damage may still lead to systemic organ failure called biotrauma [6]. Mechanical strain caused by ventilators has been shown to cause cell signaling, leading to inflammation [7]. It has also been shown that the inflammation depends on the mode of MV [8,9].

Several studies have been conducted to model air pressure on lung airway wall with diseases, such as tumors [10,11] and chronic obstructive pulmonary disease [12,13]. Fluid–solid interaction modeling using the Finite Element Method (FEM) has been employed by Koombua, *et al.* [14] to study airflow characteristics and stress distribution on airway walls. The results in [14] were used in [15] and [16] to analyze stress and strain distribution occurring on a more detailed model of tissue. However, the previous studies did not employ a full breathing cycle. The complex dynamics of lung tissue environment are still being investigated, and, although significant research has been done, there are still unanswered questions with regards to the transmission of mechanical forces into lung tissues resulting from MV. Even though there have been many studies investigating the effects of long term ventilation with respect to lungs, the connection between the global deformation of the whole lung and the strains reaching the lung tissue has not been studied. Capturing real-time progression of airway tissue stretch during breathing is still difficult to assess experimentally. Currently, there is a lack of data on real-time stretch progression of airway tissue during MV.

In this study, a scheme for studying real-time progression of airway tissue stretch is proposed. The scheme only incorporates fundamental dynamics of airway tissue structure to save on the computational cost of simulation. The scheme consists of two steps. First, airflow through a lung airway bifurcation (Generation 4–6) is modeled using Computational Fluid Dynamics (CFD) to obtain air pressure during MV breathing with a flow rate of 35 L/min, as a follow up on results from a previous study [17]. Next, the transient air pressure was used in structural analysis to obtain mechanical strain experienced by the airway tissue wall. Several simulations were carried out to investigate the stress/strain environment within the airway lung tissue, and the results are presented and discussed.

## 2. Materials and Methods

### 2.1. Airway Tissue Model

Human lung consists of bifurcating airways that begin at the trachea. The trachea is designated as Generation 0. The generation numbers are then counted from bifurcating airways that grow after the trachea. The total number of generations in the human lung is 23. In this study, airway bifurcation of Generation 4–6 is considered.

**Figure 1:**
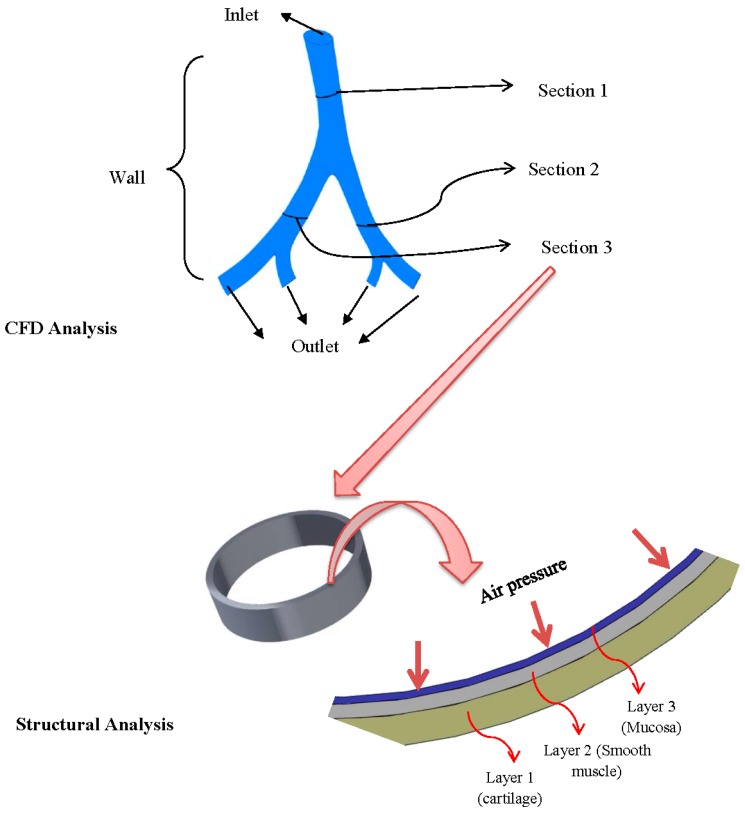
Multi-level analysis adopted for transient mechanical response of airway lung tissue. **Top**: model and boundary condition used for fluid flow analysis. **Bottom**: model for mechanical analysis.

A three-dimensional model of lung bifurcation Generation 4–6 was built based on approximation of a realistic human lung model. For the mechanical response analysis, the model was based on airway representation as discussed by Kamm [18]. The upper lung airway (Generation 1–8) is composed of three major layers based on the composition of each layer: mucosa, sub-mucosa and the area outside of the submucosa [19]. The submucosa includes the smooth muscle tissue. The area outside the submucosa consists of cartilage-fibrous layer and adventitia. In this study, a slice of airway model is considered for simplicity. The airway is assumed to stretch in radial and axial directions. The structure of the airway was simplified into a basic fundamental structure to capture the main dynamics of the airway. Figure 1 show the lung bifurcation along with the tissue layer model used in this study. Each layer is named as shown in Figure 1 for later discussion.

### 2.2. Computational Models and Boundary Conditions

In this study, two separate models were investigated using multi-level modeling. First, airflow through a lung airway bifurcation (Generation 4–6) is modeled using Computational Fluid Dynamics (CFD) to obtain air pressure (airflow model). Next, the transient air pressure was used in structural analysis to obtain mechanical strain experienced by the airway tissue wall (airway deformation model).

#### 2.2.1. Airflow Model

The momentum of airflow through the lung bifurcation was assumed to follow the Navier-Stokes equations:

Conservation of mass:

(1) $$\frac{\partial \rho }{\partial t}+\nabla .\left(\rho v\right)=0$$

Conservation of momentum

(2) $$\rho \left(\frac{\partial v}{\partial t}+v.\nabla v\right)=-\nabla p+\nabla .\left(\mu \left(\nabla v+{\left(\nabla v\right)}^{T}\right)\right)$$

where ρ is fluid density, v is velocity and p is pressure. The viscous force term in Equation (2) is in the form of incompressible viscous flow, which is the assumption of flow in airway Generation 4–6. The domain for the equations was the solid model as shown in Figure 1. Boundary conditions for Equations (1) and (2) were as follows: a velocity boundary conditions was imposed at the inlet (“top” part of airway as in Figure 1) and a pressure outlet boundary conditions at the four outlets (“bottom” part of airway as in Figure 1) assuming flow exits the bifurcation unobstructed. No-slip boundary conditions are imposed at the walls. For the pressure and velocity conditions occurring in the lung geometry, the flow is assumed to be incompressible. A rectangular MV flow rate waveform was considered in this study. A model was developed to approach the rectangular MV waveform [17,20]. The wave was modeled as a function of airway area and generation number. The equations are as follows:

(3) $$v\left(t\right)=\frac{Q}{A\;2^{N}-1}$$

(4) $$v\left(t\right)=TV.\frac{e^{-\left(t-t_{i}\right)/\tau }}{A\;2^{N}-1}$$

where $t_{i}$ is total inspiration time, *N* is generation number, *A* is cross section of airway under consideration (in this case, the airway Generation 4 model), Q is averaged flow rate, *TV* is tidal volume of lung bifurcation, and τ is ratio of tidal volume and flow rate. The flow rate is equal to the ratio of tidal volume and inspiration time. Equation (3) was used as an approach for studying inspiration airflow, and Equation (4) was used to assess expiration airflow during MV breathing. The analysis was carried out for MV breathing with a flow rate of 35 L/min, following [17]. Hence, to maintain the flow rate, the tidal volume was chosen to be 14 mL, and inspiration and expiration times were chosen to be 0.4 s and 1.6 s, respectively.

Figure 1 shows three parts of the airway wall that are designated as Section 1, Section 2 and Section 3. Section 1 is the biggest airway in the model, followed by Section 3 and Section 2. Pressures along these sections were obtained for a subsequent airway wall mechanical response analysis.

#### 2.2.2. Airway Deformation

The lung airway tissue was assumed to be an elastic material. The stress developed in the tissue by pressure applied on it was modeled using the Cauchy momentum relationship:

(5) $$\frac{\partial \sigma_{ij}}{\partial x_{j}}=\rho \frac{\partial^{2}u_{i}}{\partial t^{2}}$$

The constitutive equation is a linear elastic material as follows:

(6) $$\sigma_{ij}=E\epsilon_{ij}$$

where σ is stress tensor, ϵ is strain, *u* is displacement, *E* is elastic moduli, *t* is time and ρ is mass density. In this study, a cylindrical coordinate was used to define direction. Hence, the subscript refers to radial (r), rotational (θ) and (in three-dimensional case) axial (z) directions, respectively. The domain of Equation (5) is a slice of lung airway as shown in Figure 1. Radial contraction by smooth muscle is the dominant type of contraction when the lung airway inhales air. In this study, we are interested in the fundamental dynamics of the airway. Hence, the thickness of the model was determined to be one-third of its diameter to ensure the radial response is dominant over axial ones.

The diameters were chosen to approach three sections in the CFD analysis. These diameters are: 2.5 mm for Section 1, 1.86 mm for Section 2 and 1.45 mm for Section 3. The tissue model consists of three layers as shown in Figure 1. The thickness of layers for each section is shown in Table 1. The layers were modeled as an isotropic material. The Young’s moduli are 75 KPa for smooth muscle and cartilage layer, and 115 KPa for mucosa layer. Poisson’s ratio for all three layers is 0.45. The density is assumed to be 1365.6 kg/m^3^, after the work of Koombua and Pidaparti [14]. Isotropic material relation was used in this study since the focus of our study was to analyze the basic dynamics (axial and radial structural response) of the tissue. A recent study by Weed *et al.* [21] also shows evidence of isotropy at least in lung parenchyma. Furthermore, Xia *et al.* [22] described in their work that an anisotropic effect appears when tissue strains reached 20%. In this study, small strain assumption was used for the tissue analysis. To compare the linear isotropic material relationship with non-linear ones, two non-linear material relationships were applied. The Neo-Hookean material relationship was used with parameters as outlined in [15]; *C*_10_ = 0.56 MPa, μ = 1.12 MPa and incompressibility parameter, *d* = 2.14 × 10^−8^. Ogden material model was also used, based on measurement by Zeng, *et al.* [23]. The parameters were as follows; μ_1_ =177.93 Pa, *A*_1_ = 5.0424, incompressibility parameter, *D_1_* = 0.

**Table 1:** Material model for each layer of the airway tissue.

Layer Name	Layer Number	Thickness at Each Section		
		Section 1	Section 2	Section 3
Mucosa	Layer 1	66.56 µm	49.59 µm	38.61 µm
Smooth Muscle	Layer 2	31.95 µm	22.17 µm	18.53 µm
Cartilage	Layer 3	15.29 µm	11.37 µm	8.86 µm

The boundary conditions were as follows: the model was subjected to transient pressures on the inner surface of the airway model. The transient pressures are obtained from airflow simulation described in the previous section. The displacement in the circular direction was constrained, following the assumption described above. The slice of tissue is supposed to be a part of the larger airway. It was assumed that one side of the slice gave better support than another. Hence, one side’s face was constrained in the axial direction.

#### 2.2.3. Numerical Solution to the Models

The model equations were all solved by Finite Element Analysis through ANSYS v.14 software. Both models were discretized using ANSYS meshing module. Fluent component of ANSYS was used to solve Equations (1) and (2) with boundary conditions mentioned earlier. A User-Defined Function (UDF) code was built for generating velocity inlet boundary conditions as expressed in Equations (3) and (4). The fluid domain was discretized with 428,834 tetrahedral elements. ANSYS Mechanical was used to solve Equation (7). The tissue domain was discretized with 277,440 tetrahedral elements.

## 3. Results and Discussion

### 3.1. Airflow Simulation

**Figure 2:**
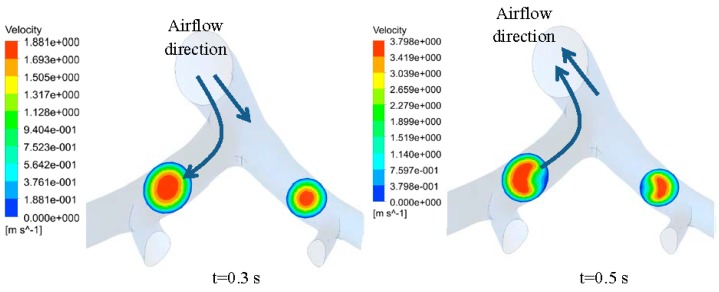
Velocity contour on bifurcation of Generation 5 lung airway, showing tendency to form horseshoe pattern.

By applying CFD analysis to lung bifurcation 4–6 using the approach described above, velocity field and air pressure inside the lung airway were obtained. Figure 2 shows contour of velocity in the immediate bifurcation after the inlet of Generation 5 airway. It can be seen from Figure 2 that the velocity patterns during inspiration and expiration are different. The velocity patterns presented in Figure 2 followed a horseshoe pattern, as previously shown in studies by Sul *et al.* [24] and Isabey and Chang [25]. This finding provides preliminary validation that the present analysis is reasonable for estimating the fluid dynamics characteristics.

**Figure 3:**
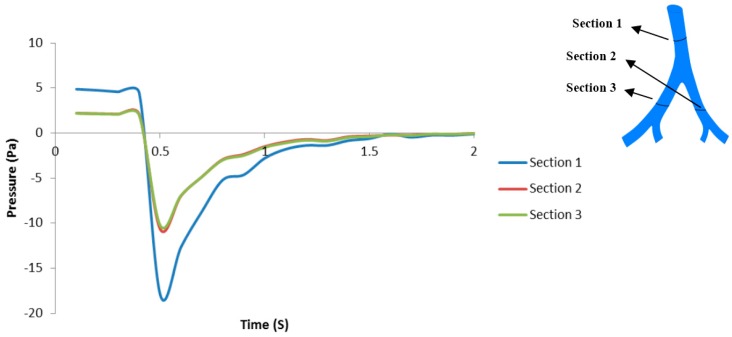
Average transient pressure for MV breathing in Section 1, Section 2 and Section 3, obtained from airflow simulation.

The average pressure on three different regions of the bifurcations (Section 1, Section 2 and Section 3) is presented in Figure 3 in order to see how the MV air pressure affects the tissues. It can be seen from Figure 3 that the pressure variation with time follows the MV velocity waveform. Also, the pressure is higher in Section 1 in comparison to other bifurcation locations (Section 2 and Section 3).

**Figure 4:**
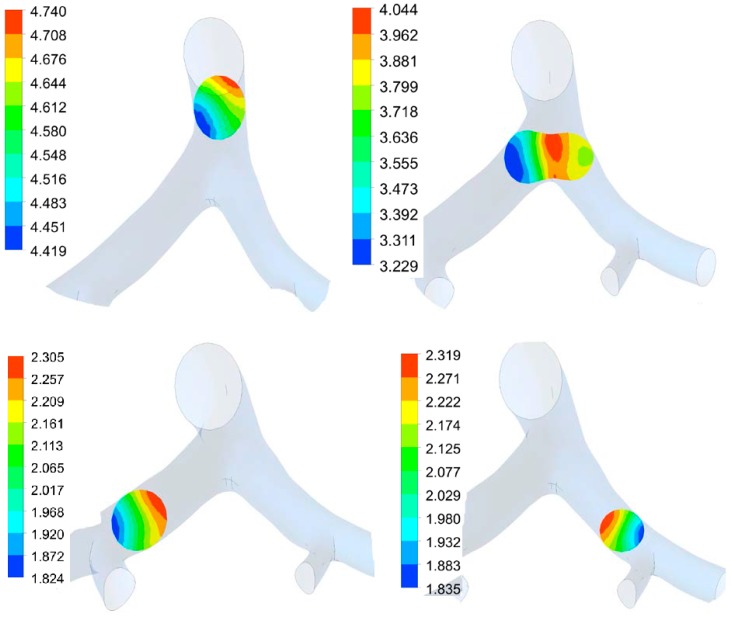
Pressure distribution in lung airway at several locations along airway at *t* = 0.3 s (inspiration) on four section, including junction. Color legend shows pressure value range in Pascal.

**Figure 5:**
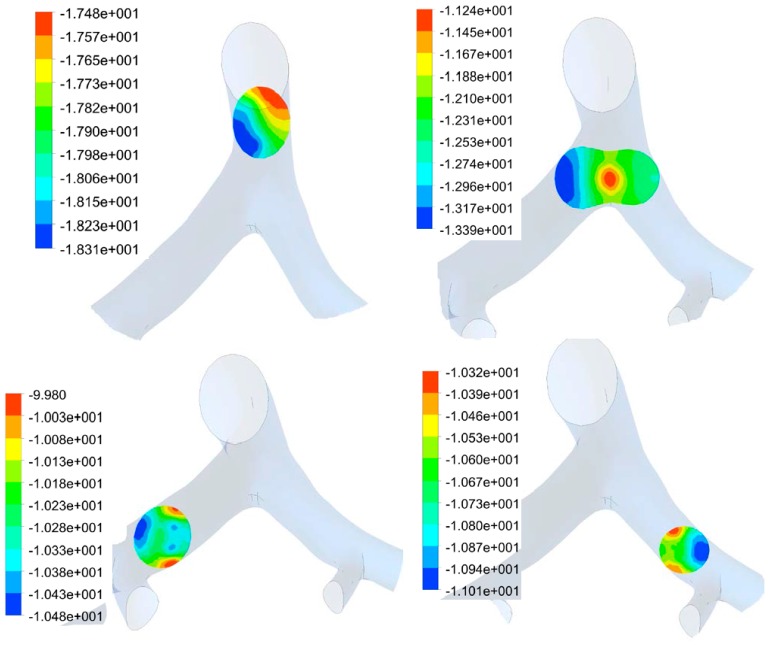
Pressure distribution in lung airway at several locations along airway at *t* = 0.5 s (peak expiration) on four section, including junction. Color legend shows pressure value range in Pascal.

The pressure along the airway at several locations at various times during inspiration and expiration is shown in Figure 4 and Figure 5. At the bifurcation junction, the pressure is concentrated in the center of the junction. In this area, the pressure on the left side of the junction is higher in value than on the right. The variations of pressure in all these location are shown to be less than 3 Pa. These results suggest that the MV air pressure is non-linear along the radial path of the airway. There are concentrations of pressure on the three junctions during inspiration.

### 3.2. Transient Mechanical Response

#### 3.2.1. Mesh Convergence Study

**Figure 6:**
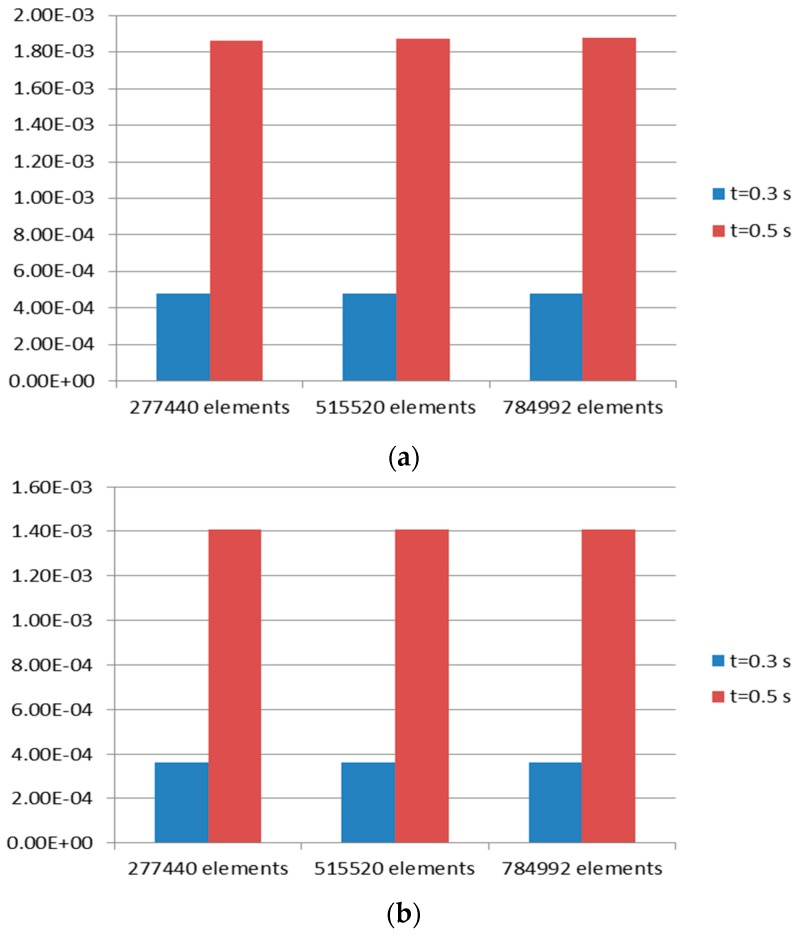
Mesh convergence test for tissue domain. Von Mises strain at two time values, *t*, was taken as criteria. (**a**) Maximum strain; (**b**) minimum strain.

Mesh convergence test was carried out before determining that the number of elements as mentioned in Section 2.2.2 was sufficient for this study. The tissue domain was discretized three times with an increasing number of elements as described in Figure 6. The Von Mises strains at two time values were chosen for comparison. The two time values will also be used in later sections. The inspiration time value of 0.3 s, which is close to start of expiration, was chosen. The second time value chosen was t = 0.5 s, which is the amplitude of the MV wave.

Figure 6 shows maximum and minimum Von Mises strain at these time values. It can be seen clearly from Figure 6 that the Von Mises strain has reached convergence at first discretization with 277,440 elements. As shown in Table 2 and Table 3, a solution obtained with 784,992 elements was named Case 3. Data presented in Table 2 and Table 3 also show that the differences between solution (Von Mises strain) of Case 3 and two other cases are negligible. Thus, discretization with 277,440 elements was chosen for this study.

**Table 2:** Difference of maximum Von Mises strain between solution of Case 3 and two other cases (t is time value).

Case Name	Number of Elements	*t* = 0.3 s	*t* = 0.5 s
Case 1	277,440	3.45 × 10^−0.06^	1.40 × 10^−0.05^
Case 2	515,520	1.24 × 10^−0.06^	5.00 × 10^−0.06^
Case 3	784,992		

**Table 3:** Difference of maximum Von Mises strain between solution of Case 3 and two other cases (t is time value).

Case Name	Number of Elements	*t* = 0.3 s	*t* = 0.5 s
Case 1	277,440	−1.00 × 10^−0.08^	−1.00 × 10^−0.07^
Case 2	515,520	0.00	0.00
Case 3	784,992		

#### 3.2.2. Airway Strains

**Figure 7:**
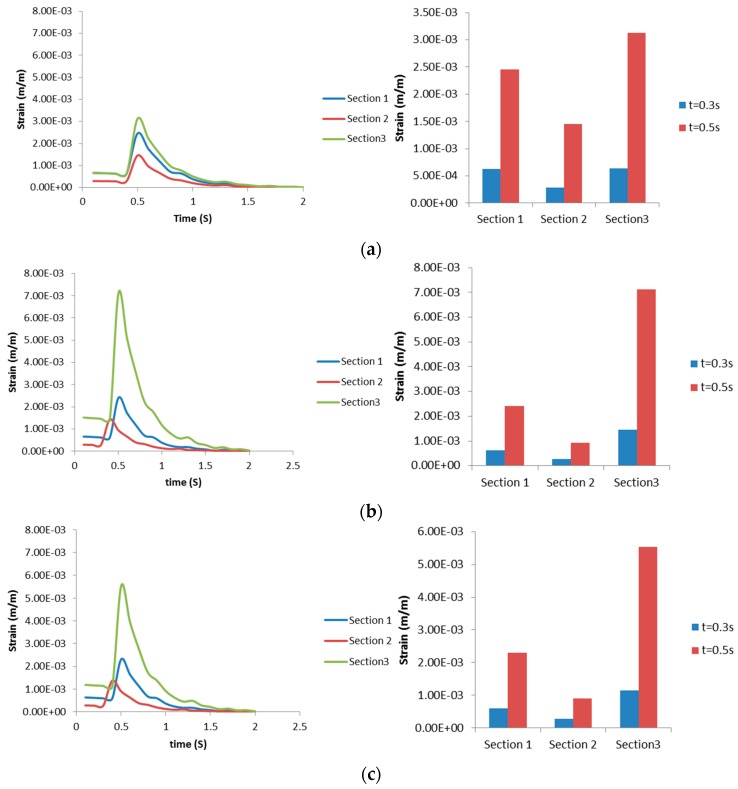
Maximum Von Mises strains in (**a**) Layer 1; (**b**) Layer 2; and (**c**) Layer 3. Right column shows strain progression over time, left column captures the Von Mises strain at a specific time for a clearer comparison.

Figure 7 shows maximum Von Mises strain over the domain representing each bifurcation generation. Generally, the highest equivalent strain occurred in Section 3, compared to other sections of bifurcation. The highest equivalent strain is 0.711% and occurred in Layer 2 (smooth muscle) of Section 3 (mucosa). The maximum equivalent strain distribution from Section 3 also showed a significant difference (up to 0.24%) between Layer 1 (cartilage) and 3 (mucosa), compared to the same distribution between Layer 1 and 3 at Section 1 and Section 2 (up to 0.015% and 0.0088%, consecutively). 

**Figure 8:**
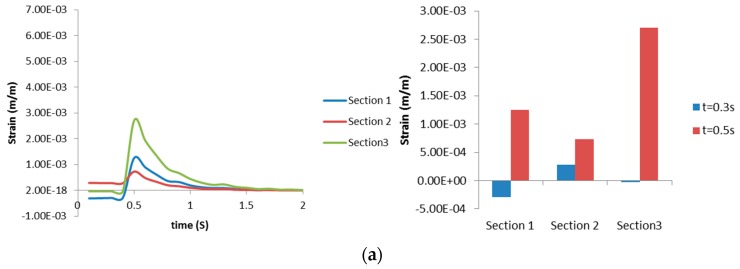

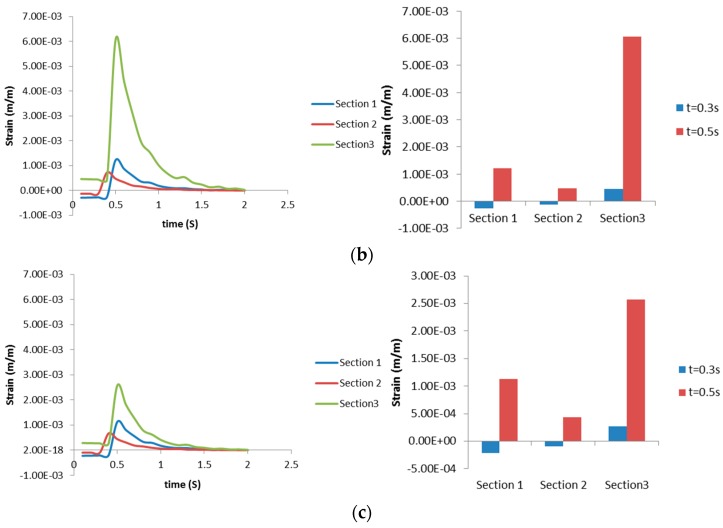
Maximum radial strains occurring in (**a**) Layer 1; (**b**) Layer 2; and (**c**) Layer 3. Right column shows strain progression over time, left column captures the Von Mises strain at specific time for a clearer comparison.

**Figure 9:**
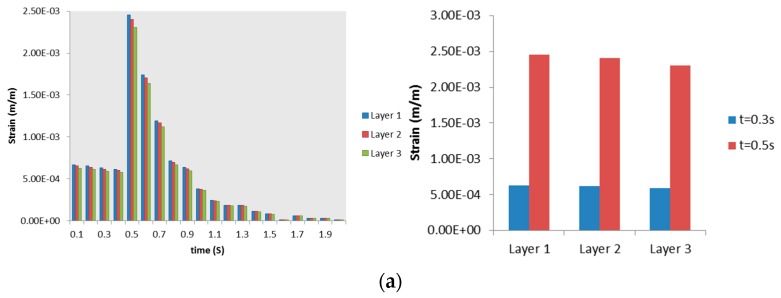

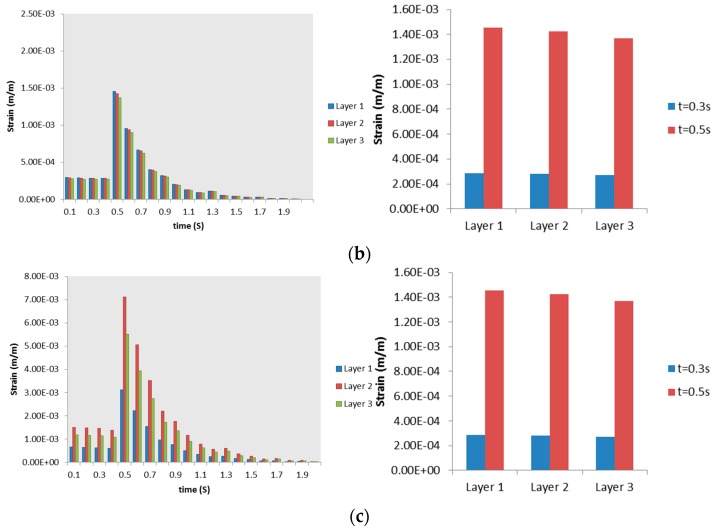
Maximum Von Mises strains occurring in (**a**) Section 1; (**b**) Section 2; and (**c**) Section 3. Right column shows strain progression over time, left column captures the Von Mises strain at specific time for a clearer comparison.

**Figure 10:**
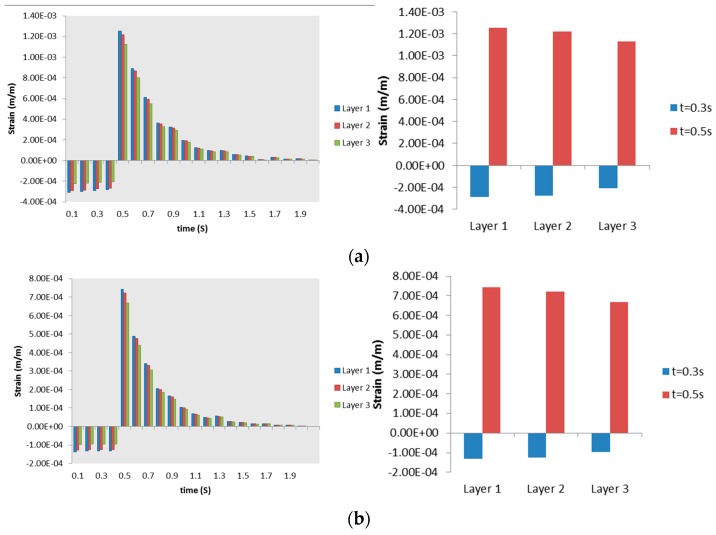

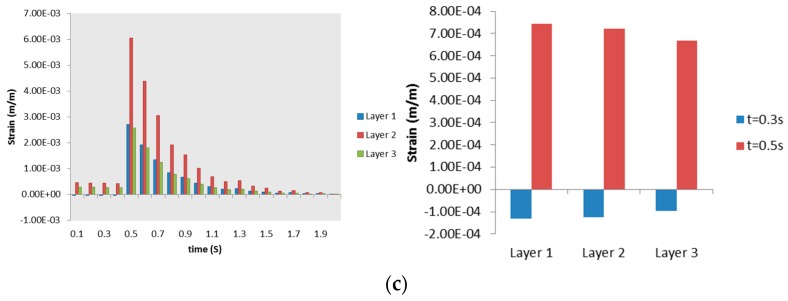
Maximum radial strains occurring in (**a**) Section 1; (**b**) Section 2; and (**c**) Section 3. Right column shows strain progression over time, left column captures the Von Mises strain at specific time for a clearer comparison.

The maximum radial strains obtained from the structural analysis in each of the sections (Section 1, Section 2 and Section 3) are shown in Figure 8. It can be seen from Figure 8 that the radial strain is higher in Layer 2 at the location of Section 3. This trend is also true for axial strains (not shown). Also, the direction of radial strain can be different for each layer at the same time, revealing unusual dynamics between layers. Figure 9 shows there is no significant difference of Von Mises strain distribution between Layers, compared to strain distribution between Sections in Figure 7. In both Figure 10a,b, the maximum radial strains in Section 1 and Section 2 during inspiration are in the negative direction. In Figure 10c, it is apparent that the direction of maximum radial strains is positive in Layers 2 (smooth muscle) and 3 (mucosa), while maximum axial strain in Section 3 is always in the positive direction during inspiration and expiration. The results presented in Figure 7, Figure 8, Figure 9 and Figure 10 illustrate the distribution and magnitude of strains in different layers of the tissue during inspiration and expiration. This is very important as the tissue response depends on the composition of the layers making up the tissue.

**Figure 11:**
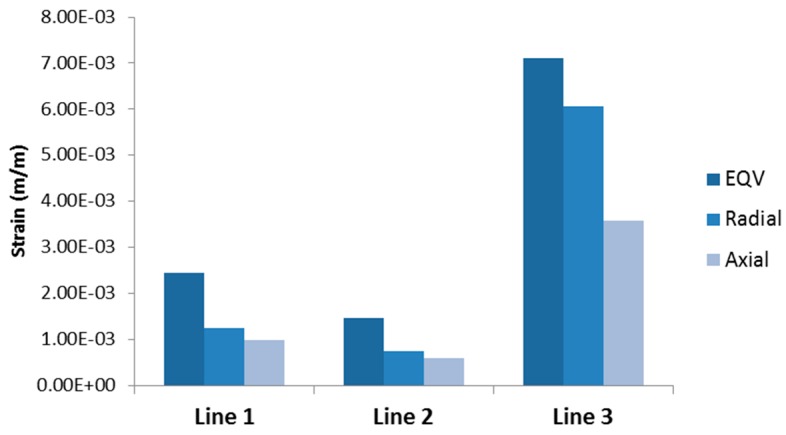
Maximum strains at peak expiration (0.5 s). Von Mises strain in Section 3 is 4.89 times and 2.9 times higher than in Section 2 and Section 1, respectively. Radial strain in Section 3 is 8.15 times and 4.83 times higher than in Section 2 and Section 1, respectively. Axial strain in Section 3 is 6.04 times and 3.6 times higher than in Section 2 and Section 1, respectively.

Figure 11 summarizes strains obtained for each section representing Generation 4–5 at time 0.5 s. Radial strain dominates over axial strain following our assumption as discussed in Section 2.2.2. It can be clearly seen that von-Mises strain in Section 3 (Generation 5 to the left of bifurcation defined by the illustration) is 2.9 times and 4.89 times the strains in Section 1 and Section 2, respectively. The radial and axial components of the strain in Section 3 are also higher than in Section 1 (8.15 times for radial and 6.04 times for axial) and Section 2 (4.83 times for radial and 3.6 times for axial).

**Figure 12:**
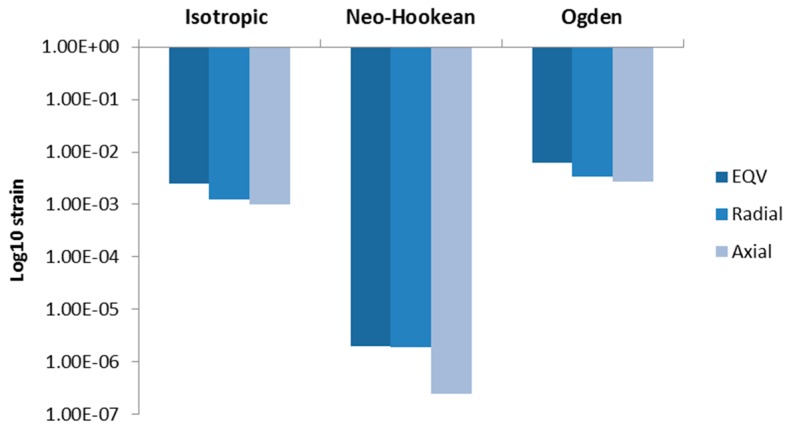
Comparison of maximum strains with different material model at peak expiration (*t* = 0.5 s). The plot is log_10_ plot to exaggerate the proportion of strains from Neo-Hookean material model.

Figure 12 shows a comparison of strain occurring at Generation 4 (Section 1) at the same time as in Figure 9 (*t* = 0.5 s) by using different material models including non-linear, Neo-Hookean and Ogden models. The results were presented in a Log_10_ plot. The Neo-Hookean model produced the smallest range of strains. The von-Mises strain from assuming Neo-Hookean material is 8.16 × 10^−4^ smaller than that obtained from the linear isotropic material relationship. However, the von-Mises strain from applying the Ogden material relationship is 2.48 times higher than a linear isotropic relationship. Since non-linear deformation was ignored in this study, and only basic response (radial and axial strains) was the focus of this study, there was no need to continue using non-linear material relations in this case. However, the Ogden material parameters used in this study may lead to results that better match the linear isotropic ones.

### 3.3. Discussion

Figure 3 shows the average transient air pressure at the three locations of the airway. The average air pressure at Section 2 and Section 3 (both are Generation 5 of airway) are almost the same, while Section 1 (Generation 4) shows the highest average air pressure distribution during inspiration and expiration. It can be concluded that the air pressure reduces as the air flows through higher generations of airway, as expected. However, this pressure could cause different dynamics at the tissue level that contain three layers. The simplified tissue model used in this study shows that location in one of the Generation 5 (Section 3) bifurcations generates more mechanical response than the location at Generation 4 (Section 1) and Generation 5 (Section 2), as shown in Figure 7 and Figure 8. The Von Mises strain (as mechanical response measure) occurring in one bifurcation of Generation 5 (Section 1) is about four times higher than in another bifurcation of the same generation (but with different bifurcation).

The only physical difference between the two Generation 5 sections here is the radius (and consequently the thickness of each layer); generation in Section 2 (1.86 mm in diameter) is about 0.8 times bigger than in Section 3 (1.45 mm in diameter). When subjected to almost similar pressure cycles, these two sections behaved differently. The mechanical response can be up to four times higher for the bigger bifurcation as described in the previous section. The material properties of the two sections in Generation 5 are the same, and the loadings are similar as shown in Figure 3, however, the thickness is different. This suggests sensitivity to geometrical features. Further study is needed to determine factors that caused different mechanical responses in the same generation in this study.

The importance of investigating strain as a mechanical response lies in its ability to detect/assess inflammation and cell signaling. It is known that cyclic mechanical strain induces varied cell signaling, such as shown in [26]. Mechanical strain may change the properties of the airway [27] and lead to inflammation. The mechanism of inflammation associated with MV breathing is not well established and there are different proposed mechanisms, such as discussed in [28] and [7]. Additionally, some authors have conducted experiments to determine a strain threshold that leads to inflammation [29,30]. However, combining mechanical analysis as presented in this study with stochastic modeling of inflammation (such as [31]) may reveal important dynamics between organ level and cell level mechanics that lead to tissue injury and/or differences in airway properties.

## 4. Conclusions

In this study, a multi-level modeling scheme was employed to investigate mechanical responses caused by air pressure during MV breathing. A bifurcation at Generation 4–6 of a realistic human lung model was considered for this study. ANSYS software package v.14 was used to solve equations for modeling airflow and structural responses of the airway. One section in Generation 4 and two sections in Generation 5 were selected for structural analysis. Fluid flow simulation showed expected pressure distribution from higher generations to lower ones. Mechanical responses to the air pressure in three locations of bifurcation showed different dynamics between two sections at the same generation with similar transient load. Mechanical response at the same generation but with different bifurcation can differ by up to eight times (peak radial strain in Section 3 is eight times that of Section 2). This result suggests sensitivity to geometrical features. Further study is needed to quantify the sensitivity of mechanical responses to geometrical features.
